# A Silent Exonic SNP in *Kdm3a* Affects Nucleic Acids Structure but Does Not Regulate Experimental Autoimmune Encephalomyelitis

**DOI:** 10.1371/journal.pone.0081912

**Published:** 2013-12-03

**Authors:** Alan Gillett, Petra Bergman, Roham Parsa, Andreas Bremges, Robert Giegerich, Maja Jagodic

**Affiliations:** 1 Department of Clinical Neuroscience, Centre for Molecular Medicine, Karolinska Institutet, Stockholm, Sweden; 2 Center for Biotechnology and Faculty of Technology, Bielefeld University, Bielefeld, Germany; Friedrich-Alexander University Erlangen, Germany

## Abstract

Defining genetic variants that predispose for diseases is an important initiative that can improve biological understanding and focus therapeutic development. Genetic mapping in humans and animal models has defined genomic regions controlling a variety of phenotypes known as quantitative trait loci (QTL). Causative disease determinants, including single nucleotide polymorphisms (SNPs), lie within these regions and can often be identified through effects on gene expression. We previously identified a QTL on rat chromosome 4 regulating macrophage phenotypes and immune-mediated diseases including experimental autoimmune encephalomyelitis (EAE). Gene analysis and a literature search identified lysine-specific demethylase 3A (*Kdm3a*) as a potential regulator of these phenotypes. Genomic sequencing determined only two synonymous SNPs in *Kdm3a*. The silent synonymous SNP in exon 15 of *Kdm3a* caused problems with quantitative PCR detection in the susceptible strain through reduced amplification efficiency due to altered secondary cDNA structure. Shape Probability Shift analysis predicted that the SNP often affects RNA folding; thus, it may impact protein translation. Despite these differences in rats, genetic knockout of *Kdm3a* in mice resulted in no dramatic effect on immune system development and activation or EAE susceptibility and severity. These results provide support for tools that analyze causative SNPs that impact nucleic acid structures.

## Introduction

Genetic variations are used as markers to discover genes that regulate Mendelian and complex phenotypes or diseases. Genome-wide association studies (GWAS), candidate gene approaches and analyzing rare mutations are methods that have been utilized to define phenotype and disease determinants. Single nucleotide polymorphisms (SNPs) are the most common genetic variation in the human genome with approximately 1.6 million common variants currently defined [1]. Genetic variations, including coding SNPs, may also be causative of phenotypes and disease susceptibility [2]. The most frequent modes of action of causal SNPs include amino acid substitutions and alternative splicing that affect protein conformation, charge and enzymatic activity, or SNPs in regulatory regions that affect transcription [3]. Estimates from the 1000 Genome Project indicate that there are 10,000 – 11,000 non-synonymous SNPs that affect amino acid coding and 10,000 – 12,000 synonymous SNPs in the coding regions of each individual [4]. Even synonymous SNPs that do not affect the coding of amino acids are under evolutionary selection pressure as they can govern a variety of mechanisms including codon selection bias, rate of protein synthesis and mRNA stability [5].

Quantitative trait loci (QTL) mapping, expression level determination, and expression QTL analyses have been used to successfully narrow the search for genes regulating disease and developmental processes [6]. These types of studies provide a wealth of insight and knowledge into disease susceptibility and pathogenesis. However, the number of polymorphisms in the population is vast and the effect of each is unknown. We recently reported a QTL governing tumour necrosis factor (TNF) production in rats through the regulation of a multitude of immune response genes and macrophage activation phenotypes [7]. The QTL also regulates susceptibility to multiple immune-mediated diseases including experimental autoimmune encephalomyelitis (EAE) [8], collagen-induced arthritis [9], pristine-induced arthritis [10], and experimental autoimmune neuritis [11]. To identify a candidate gene we hypothesized that the regulator would control many genes in the immune system because of multiple affected genes. These assumptions led us to focus on a candidate gene in the locus, lysine (K)-specific demethylase 3A (*Kdm3a*) that affects chromatin modifications.

Chromatin is built up of nucleosomes which consist of 146 base pairs of DNA wrapped around an octet of histone proteins, two of each H2A, H2B, H3 and H4. The histone tails are available for post-translational modifications including among others methylation, acetylation, ubiquitination and phosphorylation. The combination of these modifications determines the chromatin state and regulates transcriptional activation or repression [12]. Enzymes from different protein families regulate the addition and removal of histone modification. Lysine specific demethylase 1 (LSD1) was the first catalytic enzyme that was identified to specifically remove methylation from lysine residues [13]. Jumonji C (JmjC) domain-containing family proteins also demethylate specific lysine residues but are unique in that the enzymes can remove all three histone lysine methylation levels (mono-, di- and trimethylation) [14]. Methylation of histone lysine residues can activate or repress transcription depending on the particular lysine residue that is methylated and the level of methylation [15], [16], [17].

*Kdm3a* lies at the peak of confidence interval of the QTL regulating TNF in macrophages. *Kdm3a* catalyzes the reversal of H3K9 methylation states [18]. Di- and trimethylation of H3K9 is associated with transcriptional repression [19], [20]. Recently the JmjC domain-containing family has been implicated in immune system regulation and macrophage phenotypes [21], [22], [23], [24], [25]. *Kdm3a* knockout mice have a defined metabolism and obesity phenotype, but the mice have never been examined with regards to the immune system [26]. Here we present findings of a silent synonymous SNP that creates structural nucleic acid aberrations in rats. However, *Kdm3a* does not control the susceptibility to experimental autoimmunity.

## Materials and Methods

### Ethics statement

All experiments in this study were approved and performed in accordance with the guidelines from the Swedish National Board for Laboratory Animals and the European Community Council Directive (86/609/EEC) under the ethical permits N332/06, N338/09, entitled ‘Genetic regulation, pathogenesis and therapy of EAE, an animal model for multiple sclerosis’, which were approved by the North Stockholm Animal Ethics Committee (Stockholms norra djurförsöksetiska nämnd). Animals were tested according to a health-monitoring program at the National Veterinary Institute (Statens Veterinärmedicinska Anstalt, SVA) in Uppsala, Sweden.

### Animals and care

Inbred dark agouti (DA) rats were originally obtained from the Zentralinstitut für Versuchstierzucht (Hannover, Germany) and major histocompatibility complex (MHC)-identical piebald virol glaxo (PVG) rats from Harlan UK Limited (Blackthorn, UK). Local colonies were established at Karolinska Hospital 15 years ago (DA/Kini and PVG.1AV1/Kini). *Kdm3a* knockout mice (a.k.a. *Jhdm2a* ^−/−^) were received from Dr. Yi Zhang, University of North Carolina at Chapel Hill, USA [26]. Briefly, a loxP site and a β-Geo cassette flanked by two loxP sites were introduced into the *Jhdm2a* locus (in this study referred to as *Kdm3a*). E14 embryonic stem (ES) cells (derived from 129Sv strain) having undergone homologous recombination thus having 3loxP sites were isolated using standard procedures. Chimeric mice were mated with wild-type C57BL/6J mice to generate F1 with a 3loxP allele. The F1 mice were crossed with EIIa-Cre mice in C57BL/6J background to obtain offspring with 1loxP allele (*Kdm3a*+/− mice). These mice were backcrossed to the C57BL/6J strain for five generations. *Kdm3a*−/− mice and littermate controls from an F2 *Kdm3a*+/− breeding were used in all experiments. Genotyping was performed as described previously and the knockout obese phenotype confirmed in older mice lacking *Kdm3a* [26]. Animals were bred in the animal facility at Karolinska Hospital (Stockholm, Sweden) in a pathogen-free and climate-controlled environment in polystyrene cages containing aspen wood shavings with free access to standard rodent chow and water with regulated 12-hour light/dark cycles. Heterozygous mice were bred to obtain 8- to 10-week-old littermate controls for all experiments, a time-point when both knockout and wild type mice are of equal weight.

### Tissue and cell collection

Animals were sacrificed using carbon dioxide and spleens, lymph nodes and femurs were extracted and placed in DMEM (Gibco-BRL, Grand Island, NY, USA) enriched with 5% fetal calf serum (FCS), 1% L-glutamine, 1% penicillin-streptomycin, 1% pyruvic acid (all from Life Technologies, Paisley, Scotland) and 50 µM 2-Mercaptoethanol (complete media; Gibco-BRL). Spleens and lymph nodes were mechanically separated by passing through a mesh screen with the bolus of a syringe. Splenocytes were subjected to erythrocyte lysis using 0.84% NH_4_Cl pH 7.2-7.4 (Sigma-Aldrich, St. Louis, USA) and washing with complete media (CM). Bone marrow (BM) cells from mouse femurs were collected by flushing through media with a 23-guage needle. Single-cell suspensions were prepared and resuspended in CM plus 20% FCS and 20% L929 cell line supernatant (L929 SN) to induce macrophage differentiation. Dendritic cell (DC) maturation was induced with the addition of 20% X63 cell line SN and 20% FCS. BM cells were cultured for 8 days with 2 media changes and for a further 2 days without L929 or X63 SN. Macrophages were detached by adding pre-warmed (37°C) 1x trypsin-EDTA (Gibco-BRL) and mechanical scraping. Cells were suspended in CM and counted.

### RNA, cDNA preparation and qPCR

One million cells were washed with PBS and resuspended in RLT buffer and RNA was purified using an RNeasy Mini kit (Quiagen, Hilden, Germany), according to the manufacturer protocols, including DNase I treatment. cDNA was subsequently prepared with the iScript kit (Bio-Rad, Hercules, USA). Quantitative real-time PCR was performed using a BioRad CFX384 Touch real-time PCR system with a two-step PCR protocol (95°C for 10 min. followed by 40 cycles of 95°C for 10 sec. and 60°C for 30 sec.), using SYBR Green as the fluorophore (Bio-Rad). Expression levels and melt curves were analyzed in CFX Manager software (Bio-Rad) relative to hypoxanthine phosphoribosyltransferase (*Hprt*). The primers used for SYBR Green reactions are listed in Table 1.

**Table 1:** Primer Sequences.

Name	Forward Sequence	Reverse Sequence
*Primer a*	CGCTTTTTTCACTTCAGGAGGTT	CTTGTTTGGTGTTAAGAAGCCTTCT
*Primer b*	CAGCCAACACATCCCCTTTA	GGCATAGTCAGCTGTTTTTCCT
*Primer c*	ACCAGTGGCAATGTCAACAAG	CAAGGGTGGAAGGCATTTG
*Primer d*	TGGCCTCTAGACTGCCAAACTAC	CGATCTTCTGGAGTGATCAATCC
*Hprt*	CTCATGGACTGATTATGGACA	GCAGGTCAGCAAAGAACTTAT

### Western blot

Eight million cells were washed and resuspended in 200 µl Laemmli sample buffer (Bio-Rad) and stored at −20°C. Samples were separated using SDS-PAGE. Primary KDM3A antibody (Santa Cruz Biotechnology, Dallas, USA) was applied to the membrane (1:100) followed by the anti-Goat secondary antibody (1:2000; Santa Cruz Biotechnology) and a direct conjugated HRP-β-actin antibody (1:10000; Sigma Aldrich). Detection of protein was performed using ECL-chemiluminescence (Amersham Pharmacia Biotech, Piscataway, USA) and the membranes were exposed to X-ray film (Hyperfilm ECL, Amersham Pharmacia Biotech). X-ray films were analyzed using a computerized image analysis program (Kodak 1D 3.5; Rochester, USA).

### Kdm3a cloning and Luciferase assays

*Kdm3a* sequence, 181bp (from 2303-2473bp in the transcript ENSRNOT00000045279, Rno5.0, release 70) that contains the silent SNP (position 2383), was cloned in the pRL-TK vector (AF025846; Promega, Madison, USA) using *Nhe I*. This created a fusion protein between *Kdm3a* fragment and Renilla under the TK promoter. Two Renilla plasmids were created, one with the DA sequence of *Kdm3a* and one with the PVG sequence of *Kdm3a* (that differ only in one silent SNP at position 2383). HEK293T cells were transfected with DA/*Kdm3a*-Renilla or PVG/*Kdm3a*-Renilla plasmid in 10:1 molar ratio with Firefly Luciferase vector (pLuc-TK) [27] according to manufacturer’s protocol (Effectene, Qiagen). Renilla and Firefly Luciferase were visualized using Dual-Glo Luciferase assay system according to manufacturer’s protocol (Promega). Luciferase activity was used to assure similar transfection efficiency between different reactions. It was not possible to test longer fragments which are predicted to have a larger effect on RNA structure and thus RNA folding because the fusion of larger fragments with Renilla Luciferase abolished its activity (data not shown).

### Sequencing

Details of the whole-genome sequencing of DA and PVG strains will be reported in a separate publication (Bäckdahl *et al*, under review in BMC Genomics). To summarize, genomic DNA was isolated from liver of female rats using a standard phenol-chloroform method. DNA was sequenced using mate-paired 1 kb, 2 kb and 4 kb libraries with the SOLiD System analyzer according to the manufacturers’ instructions (Applied Biosystems, Foster City, USA) in the Genomic Center, Uppsala University (Uppsala, Sweden). DA and PVG sequences were mapped against the Brown Norway (BN) reference genome using BWA [28]. The sequence used here is based on the 19X coverage. cDNA of exons 12 − 17 of the *Kdm3a* gene was amplified from DA and PVG spleen cDNA. The PCR product was purified using a MinElute Purification Kit (Quiagen) before being sent for sequence analysis (Eurofins MWG Operon, Ebersberg, Germany). Sequences were aligned using Vector NTI (Invitrogen, Carlsbad, USA) and the Ensembl database (http://www.ensembl.org) to identify genetic polymorphisms.

### Cell stimulation, ELISA and proliferation

DC and macrophages from mice were plated in 48 well plates (Nunc, Roskilde, Denmark) at a density of 200,000 cells/well. Cells were stimulated with CM alone, interleukin 4 (IL4, R&D, Minneapolis, USA) or 1 ug/mL LPS (Sigma-Aldrich) and interferon-gamma (IFN⋎; R&D) for 24 hours. SN was collected and tested for IL12p70 or TNF using enzyme linked immunosorbent assays, according to the manufacturer’s protocol (eBioscience, San Diego, USA). To determine proliferation, 2×10^5^ cells were plated in 96-well plates (Nunc) and stimulated with CM alone, 1 µg/ml anti-CD28 (in combination with 0.3 µg/ml anti-CD3 plated for 1 hr at 37°C), 1 µg/ml LPS, 1 µg/ml ConA or 50 ng/ml PMA and 5 ng/ml Ionomycin (all from Sigma-Aldrich), or 50 µg/mL recombinant mouse myelin oligodendrocyte glycoprotein (MOG). 1 µCi of ^3^[H]-Thymidine (GE Healthcare, Bucks, UK) was added to cultures after 54 hrs. Cells were harvested at 72 hrs using a Wallac Tomtec (Perkin Elmer, Waltham, USA) and isotype incorporation measured using a Wallac TriLux 1450 MicroBeta (Perkin Elmer). Triplicates were averaged and a stimulation index was calculated by dividing the stimulated average by the unstimulated average.

### Flow cytometry

Single cell suspensions were prepared from lymphoid organs and cells blocked in Fc block for 30 min before staining by fluorophore-conjugated antibodies (Becton Dickenson (BD), Franklin Lakes, USA). For intracellular cytokine staining, cells stimulated in vitro with soluble anti-CD3 were restimulated for 4 h with 50 ng/ml PMA (Sigma-Aldrich), 1 µg/ml ionomycin (Sigma-Aldrich), and 1 µl/ml GolgiStop (BD), followed by surface marker and intracellular cytokine staining according to the manufacturer's instructions. Cells were analyzed on a flow cytometer (BD).

### Exon Array Analysis

Affymetrix GeneChip Rat Exon 1.0ST arrays (Santa Clara, CA, USA) were used and described previously [29]. Briefly, biotin labeled cRNA was generated using the Affymetrix Small Sample Labeling Protocol vII (http://www.affymetrix.com). Approximately 200 ng total starting RNA was used for each sample. Labeled cRNA fragmentation, as well as array hybridization, washing and staining were performed as described in the Affymetrix GeneChip Expression Analysis Technical manual (http://www.affymetrix.com). CEL files were obtained with the Affymetrix Microarray Suite software and quality control was performed using the Affymetrix Expression Console. All files were deemed suitable for further study. GeneChips were normalized using Robust Multi-array Average (RMA) with median polish and sketch quantile parameters [30]. *Kdm3a* was visually inspected in the Gene View from the Partek Genomics Suite v6.4, release 081010 (Partek Incorporated, St.Louis, USA).

### Experimental autoimmune encephalomyelitis

Recombinant mouse MOG, amino acids 1–125 from the N-terminus, was expressed in *Escherichia coli* and purified to homogeneity by chelate chromatography. Mice were anesthetized with isofluorane (Forene, Abbott Laboratories, Chicago, USA) and injected s.c. with a 100 µl inoculum at the tail base containing 70 mg MOG emulsified 1∶1 in complete Freund’s adjuvant (CFA) containing 100 µg *Microbacterium Tuberculosis*. 200 ng pertussis toxin (Sigma-Aldrich) was injected i.p. at day 0 and day 2 post-immunization (p.i.). Mice were weighed and blindly scored daily from day 10 p.i. Clinical score was evaluated as follows: 1) limp tail; 2) hind leg paraparesis; 3) hind leg paralysis; 4) tetraplegia; and 5) moribund state or death. Mice suffering severe disease (score 4) or 25% weight loss were sacrificed in accordance with ethical guidelines. A score of 4 was assigned for the remaining days of sacrificed mice.

### Bioinformatic and statistical analysis

Data are presented as mean values +/− standard error of means. Significance was determined using a two-sided Student’s T test unless otherwise stated. qPCR efficiency was calculated within the CFX Manager software (Bio-Rad). To measure the structural impact of a SNP in RNA, it is not sufficient to look at the secondary structure of minimal free energy (MFE). Rather, the Boltzmann ensemble of potential folding options should be evaluated in total. Several methods have been suggested which compare the vector of base pair probabilities derived for the RNA sequence with and without the SNP [31], [32]. The Shape Probability Shift (SPS) measure was introduced in [33] for a different purpose – to compare different thermodynamic models. Here, we adapt it to evaluate SNP impact. Abstract shape analysis of RNA [34] partitions the folding space of an RNA molecule into disjoint shape classes. A shape is characterized by a particular arrangement of helices, irrespective of their length and sequence content. Probabilistic shape analysis [35] computes a probability for each shape that a sequence can fold into. In contrast to the probabilities of individual structures, which tend to be negligibly small, shape probabilities take on reasonably large values for the dominating shapes. For a given sequence *A* and its SNP version *B*, shape analysis will report the same shapes, but with different probabilities. The *Shape Probability Shift* between *A* and *B* is defined as:



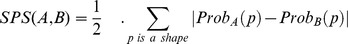



Note that the SPS is by definition a value between 0 and 1, where the extreme case of 1 would only be achieved when all shapes with positive probability for *A* have zero probability for *B* and vice versa. It can be interpreted as the overall probability mass that moves between shapes. We chose the SPS measure because of this lucid interpretation, and the extra evidence it provides by giving us best folding options within each shape. As much as a SNP can affect the folding landscape, the choice of sequence window – when the concrete, relevant part of the sequence is not known – can affect the outcome of the analysis. Therefore, we computed the SPS value for all sequence windows, up to 250bp length, and positions to overcome this caveat. For the qPCR-amplified fragment the concrete sequence is known, but for the experiment, RNA was reverse transcribed to cDNA and then amplified. Therefore, we also analyzed this sequence part using DNA folding parameters to account for the differences between RNA and DNA folding. The log-likelihood plot of the TNF-regulating QTL on rat chromosome 4 was calculated using Haley-Knott method (with sex as covariate) in 463 G10 (DAxPVG.AV1) rats using Rqtl software. The microsatellite markers locations were taken from Ensembl (Rnor5.0, release 73). The gene probabilities (represented by the frequency) and the 95% confidence interval were generated using bootstrap method (n = 1000) in Rqtl.

## Results

### Defining Kdm3a as a candidate gene

A QTL previously defined to control TNF production and an array of pro-inflammatory activation genes in macrophages was recently reported [7]. We have re-analyzed the genetic mapping using the latest and up-dated rat genome assembly, Rnor5.0. Using a bootstrap method we defined a 95% confidence interval that spans from 163.2 Mb to 164.4 Mb (Figure S1). The confidence interval contains 15 known genes which we examined for known function, SNPs, and expression levels (Table S1). Due to the regulation of a large number of genes and multiple immune-mediated diseases in previous studies we expected a ‘master regulator’. Furthermore, recent literature has implicated the JmjC domain-containing family in relation to immune system regulation [21], [22], [23], [24], [25]. This narrowed our focus to the lysine (K)-specific demethylase 3A (*Kdm3a*; ENSRNOG00000007814), which is located on rat chromosome 4 at 164282438 – 164325750 bases (www.ensembl.org, Rno5.0, release 70−71), at the peak of the QTL.

**Figure 1:**
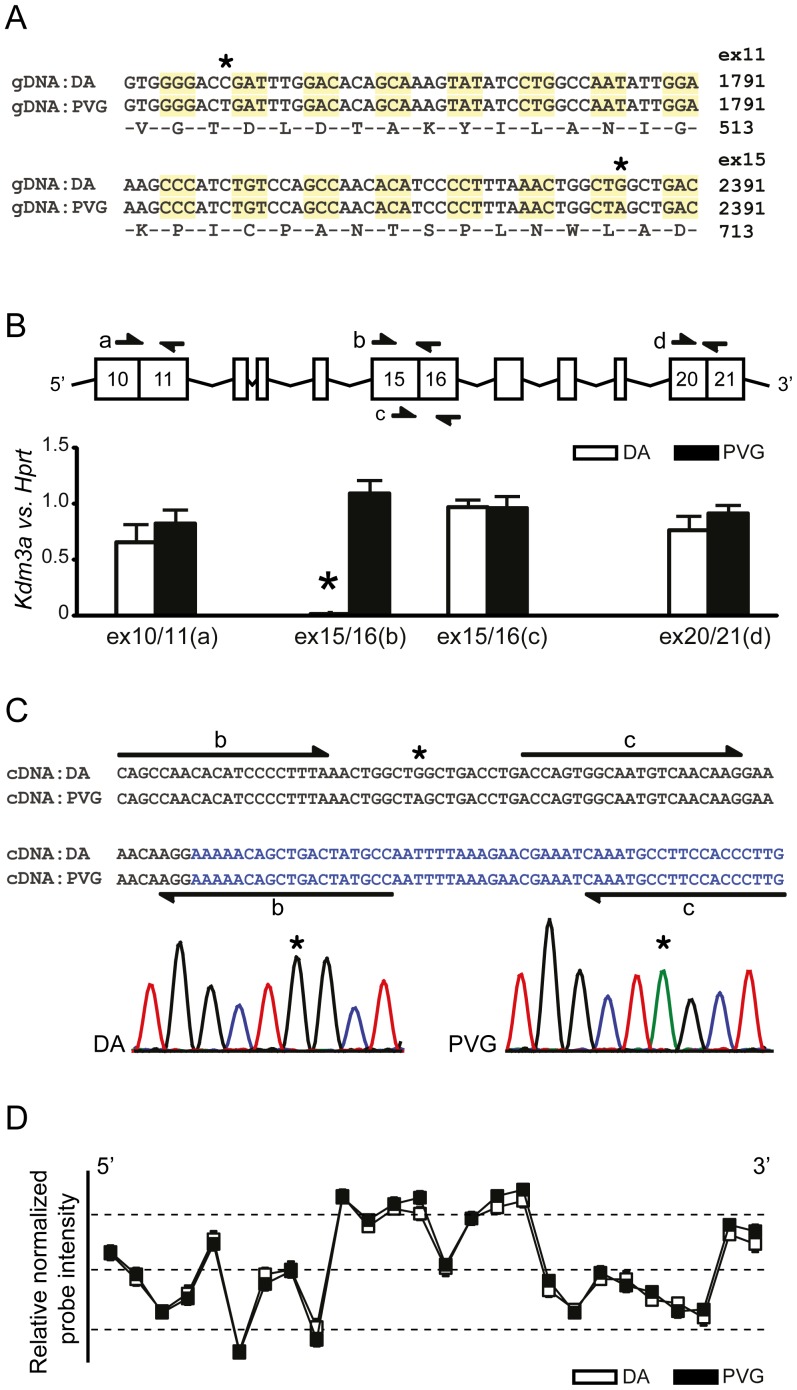
Silent synonymous SNP in exon 15 of *Kdm3a* affects RNA quantification but not alternative splicing. (A) SOLiD sequencing of genomic DNA identified two coding synonymous SNPs (asterisk) between DA and PVG strains in exons 11 and 15. (B) Quantitative PCR of *Kdm3a* in spleen tissue implicated similar *Kmd3a* levels in DA and PVG with *primers a, c* and *d* and dramatic amplification problems of *Kdm3a* in the DA strain with *primer b* (asterisk). Arrows indicate locations of primer sets in exons of *Kdm3a*. (C) Sequencing of spleen *Kdm3a* (exon 12−17) cDNA confirmed the SNP (asterisk) in exon 15 and excluded alternative splicing as a cause of amplification problems. Sequence represents part of exon 15 and exon 16 in black and blue, respectively. Arrows indicate positions of *primers b* and *c*. Sequencing chromatograms are given below; Red: T, Blue: C, Green: A, Black: G. (D) Affymetrix Exon array analysis of RNA from DA and PVG lymph nodes also confirmed lack of alternative splicing. Squares indicate exon expression derived from relative normalized intensity of all probes mapping to that exon. Probes are analyzed in an exon specific manner and show no variability between strains across the entire gene.

We examined genomic DNA (gDNA) sequence of DA and PVG rat strain. With 19X sequence coverage using SOLiD we identified two SNPs within the coding sequence of the *Kdm3a* gene (Figure 1A). Amino acid substitution was ruled out as a potential cause of regulation of the phenotype since both SNPs are synonymous. We therefore focused our attention on expression levels of *Kdm3a*. We created multiple primer pairs (*a* – *d*) to examine expression in splenocytes (Figure 1B) and macrophages (data not shown). We identified dramatic quantification problems in DA strain with *primers b* but no differences with other primer sets. The PVG sample amplified 6 – 7 threshold cycles (Ct) earlier than the DA sample. We validated these findings in other cDNA samples from spleen and alternative primers adjacent to *primers b* (data not shown). As *primers a* did not show differential expression we excluded an effect of the SNP in exon 11 within *Kdm3a*. These cumulative findings led us to speculate that alternative splicing may occur between strains due to the synonymous SNP in exon 15 of the *Kdm3a* gene.

We analyzed alternative splicing using long-range PCR on spleen cDNA followed by electrophoresis. We determined only a single band for both strains supporting no alternative splicing (data not shown). We also sequenced cDNA across exons 12−17 of *Kdm3a*. We confirmed the synonymous SNP in exon 15 but alternative splicing was not implicated (Figure 1C). Furthermore, we analyzed exon array data from lymph node cell mRNA and determined no difference in expression levels between strains and no support for alternative splicing (Figure 1D). None of the annealing probes from the chip overlap the silent SNP (www.affymetrix.com).

### Silent SNP regulates cDNA quantification

**Figure 2:**
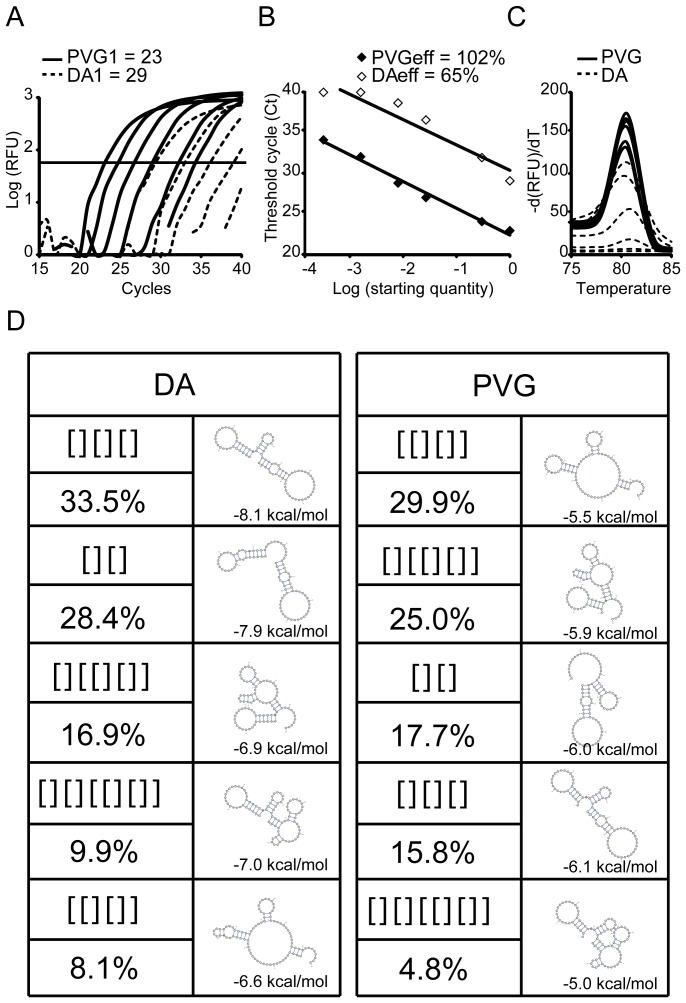
Silent SNP in exon 15 of *Kdm3a* affects qPCR efficiency due to changed cDNA structure. Six 5-fold dilutions, starting from the same amount of cDNA from DA and PVG, were subjected to qPCR with *primers b* (see Figure 1B, C). The DA SNP-containing *Kdm3a* sequence displayed higher Ct values during amplification (A), lower PCR efficiency (B) and changed melt curve pattern (C) compared to the PVG SNP-containing *Kdm3a* sequence. (D) Bioinformatics analysis of cDNA secondary structure. Shown are the most likely shapes, their probabilities, and the minimum free energy structure within each shape class together with its predicted free energy. RFU: Relative fluorescence units; eff: efficiency

As the silent synonymous SNP within exon 15 of the *Kdm3a* gene did not regulate alternative splicing we hypothesized that it may exert its effect on qPCR amplification efficiency. We created serial dilutions starting with the same amount of cDNA from DA and PVG samples to generate standard curve samples for each SNP version and compared amplification (Figure 2A). The DA cDNA amplified at a much higher Ct of 29 compared to PVG cDNA at 23, despite having the same amounts of *Kmd3a*. We evaluated amplification efficiency for each set of standards and found a strong effect of the silent SNP whereby the DA SNP-containing *Kdm3a* sequence had an efficiency of only 65% compared to 102% for the PVG SNP-containing *Kdm3a* sequence (Figure 2B). The data was further confirmed with melt curve analysis whereby the PVG SNP-containing *Kdm3a* sequence has a lower melting point than DA and a much stronger signal indicating increased amplification and quantity during PCR cycling (Figure 2C). We also confirmed these findings using a larger purified *Kdm3a* amplicon (exons 12−17) as a template for qPCR (data not shown).

We speculated that the SNP reduced amplification efficiency through altered cDNA secondary structure. We performed probabilistic abstract shape analysis of the DA and PVG variants of the 88bp amplicon using RNAshapes (http://bibiserv.cebitec.uni-bielefeld.de/rnashapes) (Figure 2D). For both DA and the PVG amplicon, simply looking at the minimum free energy (MFE) structure shows the same structure (with shape "[ ][ ][ ]"), except that one C-G base pair present DA is missing in PVG, and the free energy increases accordingly. The structural ensemble as a whole shows more difference. With DA, the representative structures of the four most likely shapes, covering 88.7% probability, all show a hairpin structure that can impede the priming site. Specifically, the first base in the amplicon is involved in this hairpin structure. With PVG, the first base is unpaired in the representative structures of four out of the five most likely shapes, the adjacent helix is shorter, and in particular, we see shape "[ ][ ]" with probability 17.7% and an MFE structure that has all bases in the primer site freely accessible. Altogether, the SNP leads to more internal structure competing with primer annealing in the DA amplicon than in PVG. This phenomenon caused the reduction in amplification efficiency and a much lower estimate of the quantification.

### Silent SNP effect on translation

**Figure 3:**
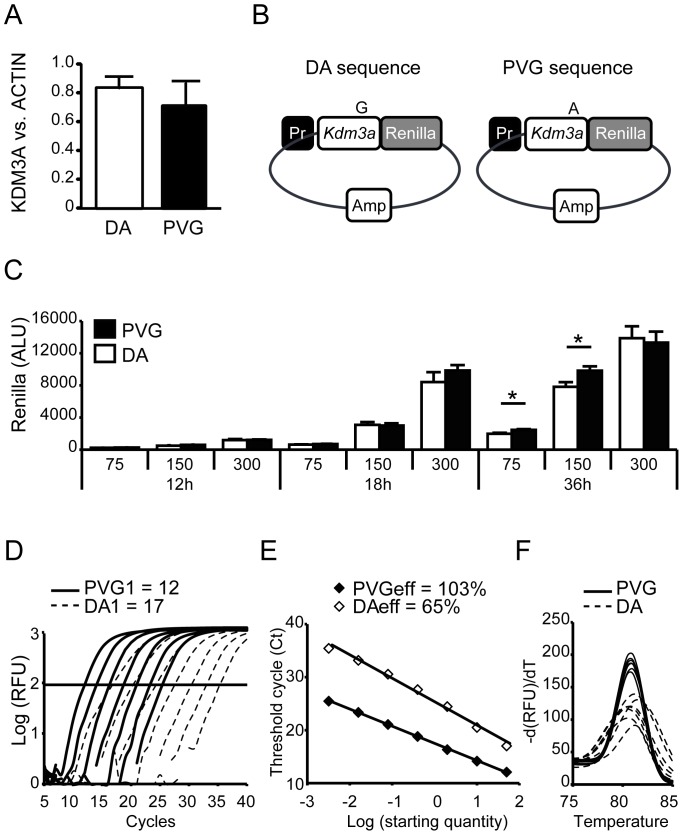
Effect of silent SNP in exon 15 of *Kdm3a* on translation. (A) Western blot analysis demonstrated equal levels of KDM3A protein in DA and PVG spleens at steady state. (B) In order to investigate the impact of the silent SNP on initiation of translation we fused in frame 181bp of the *Kdm3a* sequence from DA and PVG strains with *Renilla Luciferase* reporter gene (hereafter referred to as DA and PVG plasmids). (C) HEK293T cells were transfected with DA and PVG plasmids in different concentrations (75, 150 and 300 ng) and luminescence was measured 12, 18 and 24h after transfection. Transfection of HEK293T with DA plasmid demonstrated lower *Renilla* activity compared to PVG plasmid only under some conditions. (D, E, F) Seven 5-fold dilutions, starting from the same amount of DA and PVG plasmids (50pg/μl), were subjected to qPCR with *primers b*. The DA plasmid displayed higher Ct values during amplification (D), lower PCR efficiency (E) and changed melt curve pattern (F) compared to the PVG plasmid. Significance was determined using a Student’s T test; *: p<0.05. Pr: Promoter sequence; ALU: arbitrary luminescence units; RFU: Relative fluorescent units; eff: efficiency.

We next sought to determine if the silent SNP could regulate translation and protein levels. To evaluate this we first examined KDM3A protein levels from spleen cellular extracts using Western blot. There was no difference in the level of KDM3A protein in DA and PVG at steady state (Figure 3A). We then tested the hypothesis that variations in translation may be regulated temporally. We created a vector system whereby two inserts of 181bp, one including each version of the SNP (DA and PVG), were fused in frame with a *Renilla Luciferase* gene (Figure 3B). The vector was transfected into HEK293T cells together with a *Firefly Luciferase* reporter vector as a control of transfection efficiency. The *Renilla Luciferase* luminescence level would thus reflect the translation of the 181bp fragments and quantify the effect of the SNP on protein levels. In this system we could not demonstrate strong reproducible differences in *Renilla Luciferase* luminescence between the plasmids (Figure 3C). However, we observed a trend for lower or a significantly lower *Renilla Luciferase* luminescence after transfection with the DA-containing *Kdm3a* sequence under some conditions in several experiments.

We also verified that even the cloned silent SNP had an impact on nucleic acid structure and thus qPCR amplification similar to observations from spleen cDNA. Equal starting concentrations of the DA and PVG SNP-containing plasmids (Figure 3D) were used to generate standard dilutions and evaluated for Ct amplification. The most concentrated DA sample amplified at a Ct of 17 while PVG reached Ct at 12 (Figure 3D). We also verified the impact of the SNP on amplification efficiency; DA plasmid had an efficiency of 65% whereas PVG had 103% (Figure 3E). Furthermore, we also determined differences in melt curves similar to our cDNA results (Figure 3F).

### Silent SNP effect on Shape Probability Shift

**Figure 4:**
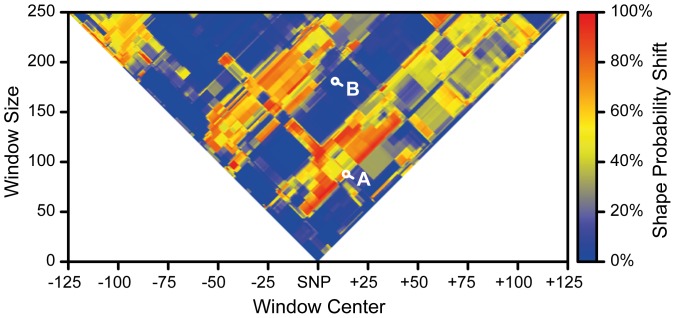
Silent SNP in *Kdm3a* affects *Kdm3a* mRNA folding. Shape Probability Shifts (SPS) between the DA and PVG versions of the *Kdm3a* transcript were presented for all sequence windows, up to 250bp length, and positions. The SPS measures the structural impact of the SNP, and it can be interpreted as the overall probability mass that moves between RNA shapes. On average the SPS is 29%, *i.e.* the SNP often affects RNA folding. Highlighted sequences are (A) the qPCR-amplified sequence (SPS 64%), and (B) the cloned sequence (SPS 1%).

To assess if the SNP can have an overall impact on RNA folding we performed Shape Probability Shifts (SPS) analysis between the DA and PVG versions of the *Kdm3a* mRNAs (Figure 4). The SPS measures the structural impact of the SNP on RNA shapes. On average the SPS is 29%, meaning that the SNP often affects RNA folding. The qPCR amplicon produced with *primers b* displayed a high probability for RNA shape change (SPS 64%) while the sequence in *Renilla* vector displayed almost no effect (SPS 1%) (Figure 4). This could explain the lack of robust effect of the cloned sequence on *Renilla-fusion* translation. Taken together, SPS data indicate that the SNP has a significant potential to affect RNA folding, which may, under some conditions, affect protein translation.

### Kdm3a knockout and immune phenotypes

**Figure 5:**
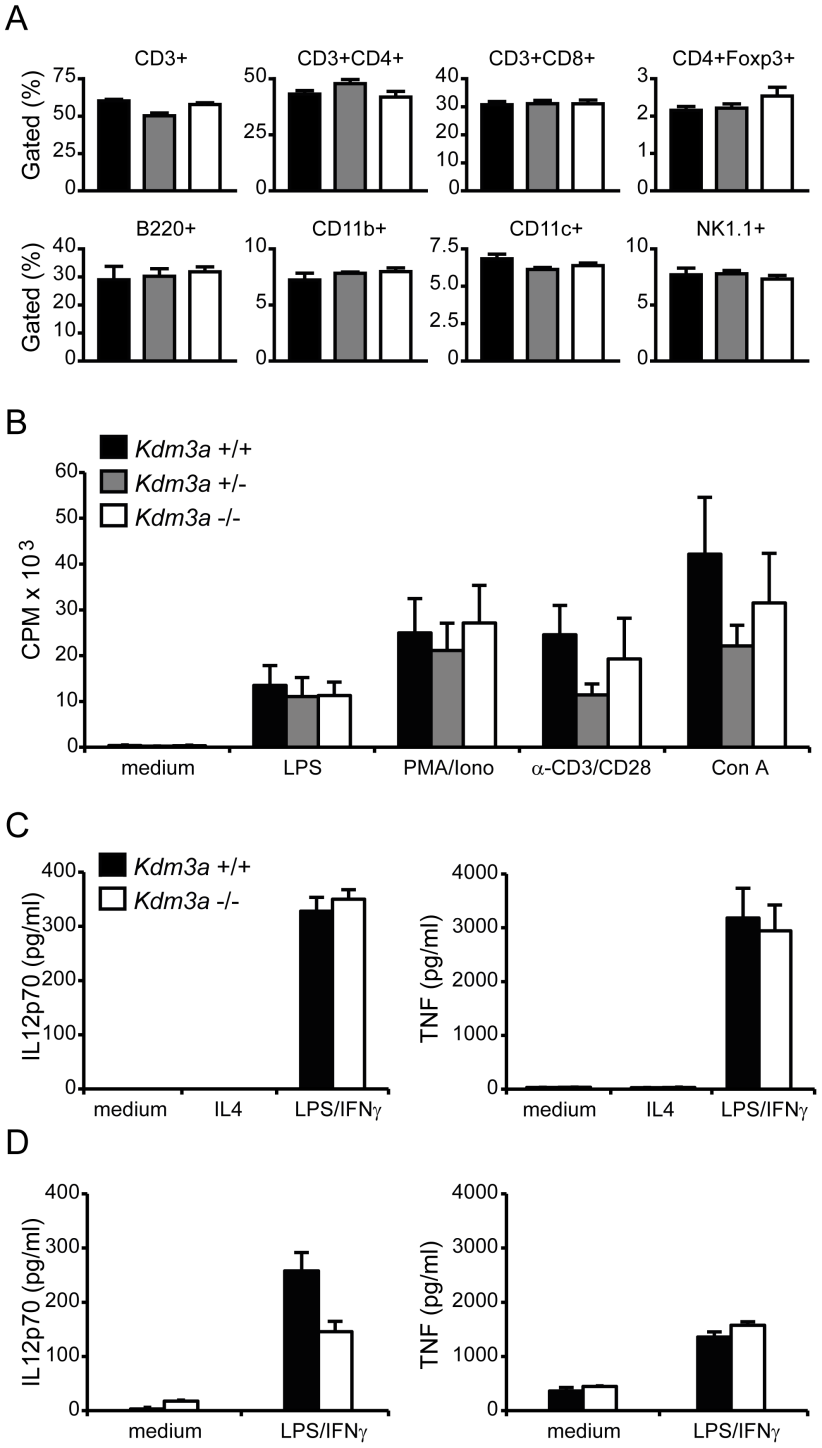
*Kdm3a* does not affect cell subsets or unspecific activation of lymphocytes, macrophages and DCs. *Kdm3a*−/−, *Kdm3a*+/− and wild type littermate control mice displayed comparable (A) frequency of all major immune cell types measured with flow cytometry in naïve spleen tissue, (B) proliferative response of spleen cells to unspecific (LPS, PMA/Ionomycin, a-CD3/CD28 and Concanavalin A stimuli) measured by thymidine incorporation 72h after stimulation. *Kdm3a*−/− and wild type littermate control mice displayed comparable production of IL12 and TNF measured by ELISA in stimulated bone marrow derived (C) dendritic cells and (D) macrophages. CPM: counts per million.

As *Kdm3a* was identified as a candidate gene regulating several immune-related QTLs, including TNF production and EAE susceptibility, we explored the effect of removing the gene on the immune system using knockout technology. *Kdm3a* ^−/−^ mice show a phenotype related to metabolic gene expression and obesity but the effect on the immune system has not been reported [26]. We observed the previously described effect on obesity in older mice (data not shown). We next investigated immune compartments relevant for EAE development. Using flow cytometry we discovered no differences in splenic immune cell populations, including T cell subsets, B cells, macrophages, dendritic cells (DCs) and natural killer (NK) cells (Figure 5A). The lymph nodes reflected similar levels of all immune cell populations between knockout and wild-type mice (data not shown). A range of stimuli, including anti-CD3/CD28, LPS, ConA and PMA/Ionomycin, produced equal proliferative responses from knockout and wild-type spleen cells (Figure 5B). The equivalent proliferative capacity was confirmed in lymph node cells (data not shown). Stimulated macrophages and DCs from knockout and wild type mice produced equal levels of TNF and interleukin 12 (Figure 5C and D). There was therefore no striking difference in the naïve immune system when *Kdm3a* was genetically removed.

### Kdm3a knockout and its effects on EAE

**Figure 6:**
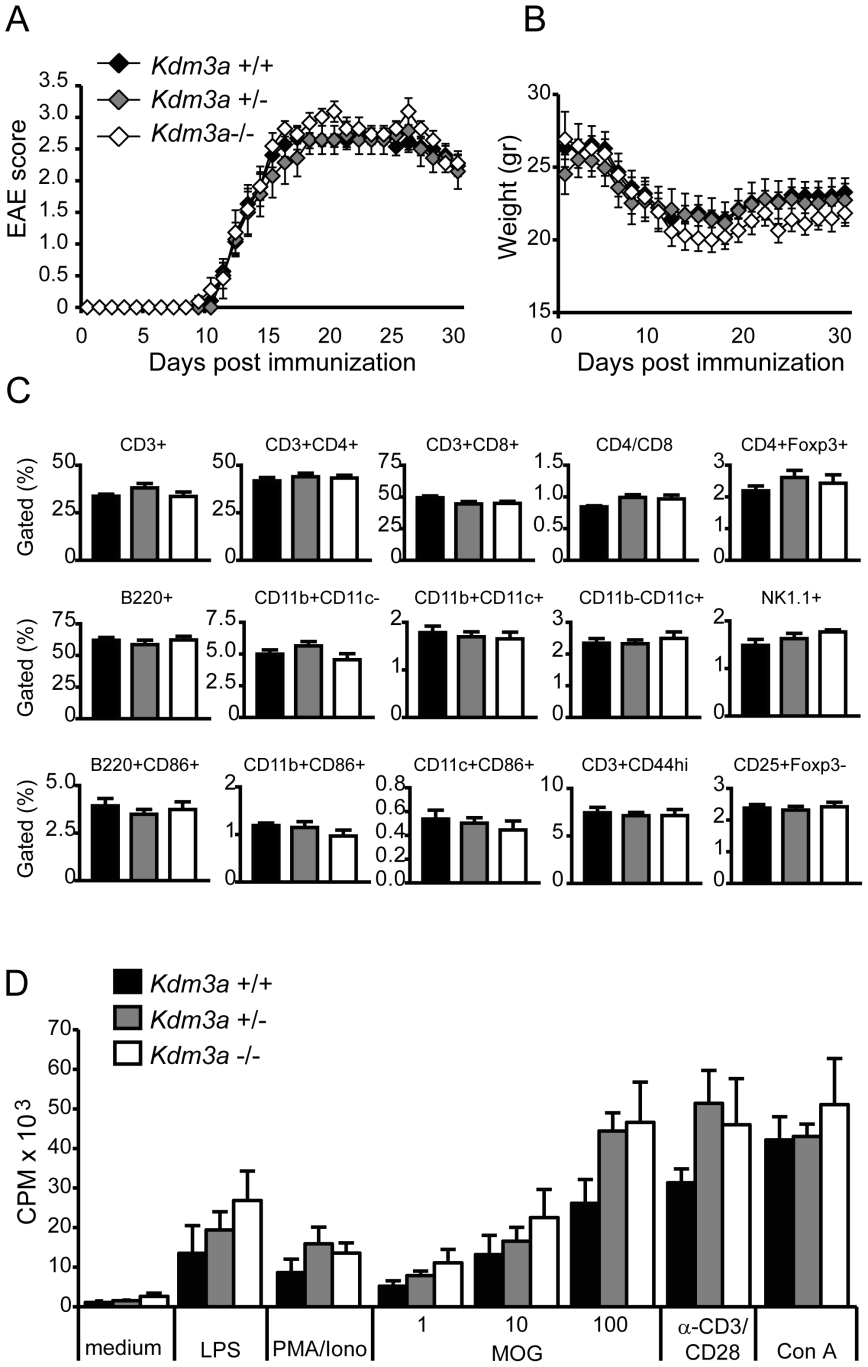
*Kdm3a* does not affect susceptibility or severity of EAE. *Kdm3a*−/−, *Kdm3a*+/− and wild type littermate control mice displayed comparable (A) clinical signs of EAE and (B) weight loss, after immunization with 70μg MOG in CFA. *Kdm3a*−/−, *Kdm3a*+/− and wild type littermate control mice displayed comparable (C) frequency of all major immune cell types measured with flow cytometry and (D) proliferative response to specific (MOG) and unspecific (LPS, PMA/Ionomycin, a-CD3/CD28 and Concanavalin A) stimuli measured by thymidine incorporation 72h after stimulation in lymph node cells day 7 after EAE induction. CPM: counts per million.

Both homozygous *(Kdm3a* ^−/−^) and heterozygous *(Kdm3a* ^+/−^) knockout mice were susceptible to experimental autoimmune encephalomyelitis and displayed equivalent incidence and severity of disease compared to wild type littermate controls (Figure 6A). Disease initiated around day 10 and peaked near day 16. As EAE is weight dependent we chose to use 8- to 10- weeks old mice, before the obesity phenotype is apparent in knockout mice. Weight was equivalent between knockout and wild type mice at disease initiation and during EAE (Figure 6B). We also investigated lymph node cell subsets and proliferative capacity during disease initiation on day 7 when cellular activation is occurring in secondary lymphoid structures. We found equal proportions of immune cells and equal activation between knockout and wild-type mice (Figure 6C). Furthermore, the proliferative responses to various stimuli were also equivalent (Figure 6D). Therefore, no differences were found in EAE initiation or disease severity between *Kdm3a* knockout and wild type mice.

## Discussion

Genetic mapping enabled identification of genetic and molecular causes of many Mendelian diseases facilitating better disease management and development of diagnostic tools. QTL mapping has led to the identification of many genetic regions regulating more complex biological processes and diseases. However, the definition of single genes and genetic variants responsible for these differences has proven to be difficult. In this study we utilized expression levels of a gene within a QTL to identify *Kdm3a* as a candidate regulating immune-mediated disease phenotypes. Our results demonstrated that *Kdm3a* has no impact on activation or susceptibility to an autoimmune disease. Nevertheless, during investigation we determined that a single silent synonymous SNP can cause a dramatic control of amplification efficiency through alterations of secondary cDNA structure. Our data add to the limited examples demonstrating importance of considering seemingly silent genetic variations.

The SNP was first identified in genomic DNA sequence analysis between strains with differing TNF production and disease susceptibility. The dramatic effect that the SNP had on gene expression quantification was not the result of alternative splicing but instead was related to variation in nucleic acid folding. A number of papers have demonstrated effects of SNPs on mRNA quantification but to our knowledge no protocols or design tools take explicitly into account impact of SNPs on secondary structure [36], [37], [38], [39]. Besides these important technical issues, the effect of SNPs on mRNA may also translate to biological functions including protein levels and function [40], [41]. Further to this concept is the issue of protein folding, which can be affected by the rate of protein translation [42], [43]. The silent synonymous SNP that we identified in *Kdm3a* did not exert robust effects on translation of *Renilla Luciferase* in our fusion system with 181bp sequence of *Kdm3a*. We did observe in nearly all experiments that the DA-sequence containing plasmid had lower *Renilla Luciferase* activity under several conditions, indicating that the SNP has a potential to affect translation. This artificial system might not mimic events that occur *in vivo* in presence of full mRNA. Indeed, our Shape Probability Shift analysis suggests a significant overall impact of the SNP on RNA folding. Thus, there is a possibility that the SNP might affect protein translation *in vivo* under some conditions. Similar results have been described in experiments on green fluorescent protein (GFP) and the tristetraprolin (TTP) genes [44], [45]. Recent study of natural selection demonstrates that synonymous SNPs are under evolutionary selection pressure implicating their functional roles [46]. Thus, our example contributes to the growing evidence of functional relevance of synonymous SNPs. Our results bring into question the accuracy of mRNA detection and quantification, especially in humans which have tens of thousands of SNPs across the coding part of the genome [4]. More importantly, tools should be developed to enable coupled studies of the role of human silent synonymous SNPs on mRNA structure and protein translation with increasing datasets and knowledge from GWAS and other studies in order to help guide our understanding of disease regulation.

*Kdm3a* was not the ‘master regulator’ of EAE that we had anticipated from our early findings. Initial mRNA detection demonstrated dramatic differences between strains with differing susceptibility to EAE and other autoimmune diseases. We tested the effect of removing *Kdm3a* through knockout technology in mice on the immune system in the context of EAE. We investigated cellular development in secondary lymphoid compartments (spleen and lymph nodes), proliferation of immune cells upon stimulation with a variety of unspecific stimuli, and response of bone-marrow derived dendritic cells and macrophages. We could not observe any robust and reproducible differences between mice lacking *Kdm3a* compared to littermate controls. Despite a lack of *Kdm3a* influence on basic immune phenotypes, we tested if *Kdm3a* had an effect on EAE as immune functions can be quite specific in the context of an autoimmune disease. Mice lacking *Kdm3a* did not differ in susceptibility to develop EAE or severity of disease compared to littermate controls. Our conclusions are based on experiments in a single background strain, C57BL/6J, and may overlook weak effects of *Kdm3a*. To examine this possibility further it would be interesting to test immune development and EAE susceptibility in another background strain.

Given the role of *Kdm3a* and histone methylation in the immune system, our findings are surprising. *Kdm3a* is a chromatin modifying enzyme with the capability to affect many processes by regulating the availability of transcription within the nucleus [18]. Histone methylation enzymes acting on specific lysine residues have been implicated in immune cell process regulation. Lipopolysaccharide tolerance is induced in part by H3K9 methylation [47]. Additionally, interleukin 2 regulation is dependent on chromatin modeling of the promoter region by H3K9 methylation state [48]. H3K9 methylation is also critical for T and B cell development [49]. Furthermore, H3K27 demethylation is critical for macrophages phenotypes and immune system activation [22], [25]. Our findings indicate that H3K9 methylation regulation may be less crucial than H3K27 methylation in the context of experimental autoimmune encephalomyelitis. KDM3A may still have an impact on the immune system in other contexts not explored in this study including infection, for example. Alternatively, a compensatory mechanism within the immune system cells may occur when *Kdm3a* is genetically removed in a classical full knock-out fashion.

In conclusion, silent SNPs provide another level of complexity in the search for causal genetic variants that control phenotypes and disease susceptibility. With the ever-increasing number of identified SNPs in human populations and knowledge from GWAS and other genetic studies we may have an opportunity to explore the data in a new way and investigate the influence of these natural variations on nucleic acid structure and protein translation.

## Supporting Information

Figure S1 **QTL on rat chromosome 4.** Log-likelihood plot of the TNF-regulating QTL on rat chromosome 4 was calculated using Haley-Knott method (with sex as covariate) in 463 G10 (DAxPVG.AV1) rats. The microsatellite markers are depicted as vertical lines on the x axis and the distance between them reflects physical location taken from Ensembl (Rnor5.0, release 73). The peak of linkage was detected on D4Mit12 at 163.8 Mb. The gene probabilities (represented by the frequency) and the 95% confidence interval, depicted as vertical red bars and the horizontal black bar, respectively, were generated using bootstrap method (n = 1000). The 95% confidence interval spans from 163.2 to 164.4 Mb.
(PDF)

Table S1 **Candidate genes in the QTL on rat chromosome 4.**
(DOC)
