# Supplementary figures and images for: A Silent Exonic SNP in Kdm3a Affects Nucleic Acids Structure but Does Not Regulate Experimental Autoimmune Encephalomyelitis
Source: PLoS One. 2013 Dec 3;8(12):e81912. doi: 10.1371/journal.pone.0081912 (PMC3849365; doi:10.1371/journal.pone.0081912)

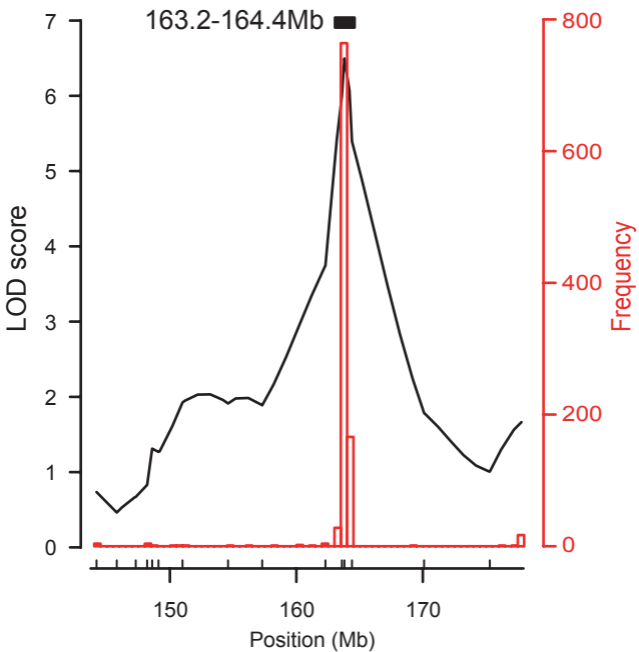

Supplement: Figure S1 — QTL on rat chromosome 4. Log-likelihood plot of the TNF-regulating QTL on rat chromosome 4 was calculated using Haley-Knott method (with sex as covariate) in 463 G10 (DAxPVG.AV1) rats. The microsatellite markers are depicted as vertical lines on the x axis and the distance between them reflects physical location taken from Ensembl (Rnor5.0, release 73). The peak of linkage was detected on D4Mit12 at 163.8 Mb. The gene probabilities (represented by the frequency) and the 95% confidence interval, depicted as vertical red bars and the horizontal black bar, respectively, were generated using bootstrap method (n = 1000). The 95% confidence interval spans from 163.2 to 164.4 Mb. (PDF) [file pone.0081912.s001.pdf]
