# Supplementary material for: A Silent Exonic SNP in Kdm3a Affects Nucleic Acids Structure but Does Not Regulate Experimental Autoimmune Encephalomyelitis
Source: PLoS One. 2013 Dec 3;8(12):e81912. doi: 10.1371/journal.pone.0081912 (PMC3849365; doi:10.1371/journal.pone.0081912)
Supplement: Table S1 — Candidate genes in the QTL on rat chromosome 4. (DOC) [file pone.0081912.s002.doc]

Table S1. Candidate genes in the QTL on rat chromosome 4

| Ensembl Gene ID | Symbol | Description | Chr | Start (Mb) | SNPs^ref1^ | Exon-arrays (p-value)^ref2^ | qPCR/FACS |
| --- | --- | --- | --- | --- | --- | --- | --- |
| ENSRNOG00000005576 | Rpia | Ribose-5-phosphate isomerase | 4 | 163,348 | 0 | ns | - |
| ENSRNOG00000006069 | Eif2ak3 | Eukaryotic translation initiation factor 2-alpha kinase 3 | 4 | 163,428 | 0 | ns | - |
| ENSRNOG00000006476 | Tex37 | Protein TSC21 | 4 | 163,507 | 0 | ns | - |
| ENSRNOG00000006491 | Foxi3 | Forkhead box protein I3 | 4 | 163,555 | 0 | ns | - |
| ENSRNOG00000006508 | Thnsl2 | Threonine synthase-like 2 | 4 | 163,753 | 0 | ns | - |
| ENSRNOG00000006675 | Fabp1 | Fatty acid-binding protein, liver | 4 | 163,840 | 0 | ns | - |
| ENSRNOG00000006776 | Smyd1 | SET and MYND domain-containing protein 1 | 4 | 163,852 | NSC:38:A/T | ns | ns |
| ENSRNOG00000007124 | Krcc1 | Lysine-rich coiled-coil protein 1 | 4 | 163,913 | 0 | 0,024 | - |
| ENSRNOG00000007129 | Cd8b | T-cell surface glycoprotein CD8 beta chain precursor | 4 | 163,964 | NSC:131:M/L | ns | ns^ref3^ |
| ENSRNOG00000007178 | Cd8a | T-cell surface glycoprotein CD8 alpha chain | 4 | 164,018 | SYC:29:Q | ns | ns^ref3^ |
|  |  |  |  |  | NSC:145:K/R | ns | ns^ref3^ |
| ENSRNOG00000028422 | Rmnd5a | Protein Rmnd5a | 4 | 164,032 | SYC:98:D | ns | - |
| ENSRNOG00000007272 | Rnf103 | E3 ubiquitin-protein ligase RNF103 | 4 | 164,178 | 0 | ns | - |
| ENSRNOG00000007356 | Chmp3 | Charged multivesicular body protein 3 | 4 | 164,228 | 0 | ns | - |
| **ENSRNOG00000007814** | **Kdm3a** | **Lysine-specific demethylase 3A** | **4** | **164,282** | **SYC:710:L** | **0,014*** | **DE** |
|  |  |  |  |  | **SYC:499:T** | **0,014*** | **DE** |
| ENSRNOG00000008481 | Reep1 | Receptor expression-enhancing protein 1 | 4 | 164,397 | 0 | ns | - |

ref1: Bäckdahl L, Ekman D, Jagodic M, Olsson T and Holmdahl R. Identification of candidate risk gene variations by wholegenome sequence of four rat strains commonly used in inflammation research. Resubmitted to BMC Genomics.

ref2: Gillett A, Maratou K, Fewings C, Harris RA, Jagodic M, Aitman T, Olsson T. Alternative splicing and transcriptome profiling of experimental autoimmune encephalomyelitis using genome-wide exon arrays. PLoS One. 2009 Nov 10;4(11):e7773.

ref3: Gillett A, Marta M, Jin T, Tuncel J, Leclerc P, Nohra R, Lange S, Holmdahl R, Olsson T, Harris RA, Jagodic M. TNF production in macrophages is genetically determined and regulates inflammatory disease in rats. J Immunol. 2010 Jul 1; 185(1): 442-50.

*probes overlap both Chmp3 and Kdm3a transcripts

0 = no SNPs

ns = not significant

- = not tested

DE = differentially expressed
